# Diaphyseal nutrient foramina in the humerus, radius, femur, and tibia bones of mixed breed dogs

**DOI:** 10.5455/javar.2021.h503

**Published:** 2021-06-15

**Authors:** Reda Mohamed, Anil K. Persad

**Affiliations:** 1 Department of Basic Veterinary Sciences, School of Veterinary Medicine, Faculty of Medical Sciences, The University of the West Indies, St. Augustine, Trinidad and Tobago; 2 Anatomy and Embryology Department, Faculty of Veterinary Medicine, Beni-Suef University, Beni-Suef, Egypt; 3 Department of Clinical Veterinary Sciences, School of Veterinary Medicine, Faculty of Medical Sciences, The University of the West Indies, St. Augustine, Trinidad and Tobago

**Keywords:** Diaphyseal, bone, dog, nutrient foramen

## Abstract

**Objective:**

The objective of this study was to determine the location, number, and direction of the nutrient foramen in the humerus, radius, femur, and tibia bones of mixed breed dogs.

**Materials and Methods:**

The humerus, radius, femur, and tibia of both (left and right) limbs of mixed breed dogs were examined in this study. The number, location, and direction of the nutrient foramina were identified. Once identified, the diameter of each nutrient foramen was measured and the site index calculated.

**Results:**

Only one nutrient foramen was identified in the humerus, radius, tibia, and right femur, while the foramen numbers ranged from one to three in the left femurs examinated. The nutrient foramen was localized on the caudal surface in the radii, femurs, tibias, and left humeri. Contrasting, however, 75% were located on the caudal surface of the right humeri and 25% on the lateral surface. The average diameter of the nutrient foramen of the humerus ranged from 0.88 to 1.00 mm, while it ranged from 1.13 to 1.25 mm in the radius. On the hind limb, the diameter of the nutrient foramen on the femur ranged from 1.2 to 1.3 mm and 0.75–1.25 mm on the tibia. The nutrient foramen was directed towards the corresponding joint in 100% of the humeri and tibias, 75% of the radii, and 60%–80% of the femurs examined.

**Conclusion:**

The anatomical data on the nutrient foramen obtained in this study will be valuable for veterinarians when diagnosing pathological bone lesions and for orthopedic surgery.

## Introduction

The vasculature plays an integral role in bone development, regeneration, and remodeling [1-3]. The development of the primary ossification center of the long bone diaphysis occurs after penetrating the nutrient artery into the cartilage. The nutrient artery enters the bone via nutrient foramina (NF) and traverseses to the medullary cavity via the nutrient canal [4]. The nutrient foramen of long bones is of clinical importance since these sites are predisposed to bone fractures [5,6]. The middle and distal parts of the diaphysis of long bones are the most frequently reported fracture sites in domestic dogs [7-9].

Damage of the leading nutrient artery of the fractured bone may lead to its poor repair or infarction [10]. The healing of bone after an insult such as a fracture is dependent on its blood supply, and herein lies the importance of the nutrient artery [11-14]. The site, size, and penetration direction of the nutrient foramen are essential anatomical characteristics for blood flow, contributing to the prognosis after a fracture [15-18]. Also, clinically, these sites are of critical importance for evaluating various pathological conditions such as developmental abnormalities and hematogenic osteomyelitis [19]. Their locations are also important in planning orthopedic procedures such as bone fracture, bone graft, and bone-implant applications [4,6,10,17,20,21] and developing new bone transplant techniques and resection [17,22].

The nutrient canal follows Bérard’s rule, which states that with the exception of some mammals and birds, the nutrient canal is directed towards the elbow joint in the thoracic limb and away from the stifle joint in the hind limb [23-25]. Wade [26] stated that the dog is the most genetically diverse domesticated animal. The vascular distribution of long bones of large breeds of dogs differs from that of small breeds [22]. The foramen index is a numerical value used to describe the location of the foramen location. The foramen index was obtained by dividing the distance between the proximal end of the bone and the nutrient foramen by the overall length of the bone [10,17,21,23,27]. Although the nutrient foramen has been studied in German Shepherds dogs [4,30], to the best of authors’ knowledge, no published reports are examining the nutrient foramen in mixed breed dogs in Trinidad and Tobago (T&T). Mixed breed dogs are common in T&T and are reared as pets and working animals, for example, for hunting and home security purposes. Knowledge of the anatomical characteristics of the nutrient foramen of these dogs’ long bones is essential for veterinarian surgeons who may be tasked with orthopedic procedures, including fracture repair and bone grafting.

## Material and Methods

The bones of the limbs of mixed breed dogs which have been stored for veterinary gross anatomy courses in the School of Veterinary Medicine, Faculty of Medical Sciences, University of the West Indies, Trinidad, and Tobago, were used for this study. Eight (8) humeri and eight (8) radii of the thoracic limbs and 10 femurs and 8 tibias of the hindlimbs were included in this study with identification of the number and direction of the bones (right and left). The anatomical parameters were measured and recorded, and the penetration direction of each nutrient foramen was identified. The numerical data were expressed as mean measurements with the [Mean ± Standard deviation (SD)]. The following data were recorded:

- Total length of the bone such that, was the distance from the greater tubercle to the condyle in the humerus, from the head of the radius to the styloid process in the radius, from the greater trochanter to the condyle in the femur and from the lateral condyle to the lateral malleolus in the tibia.
- The direction of the bones (right or left).
- The location of the nutrient foramen on the surface of the bone.
- The number of the nutrient foramen.
- The diameter of the nutrient foramen.
- The distance between the site of the nutrient foramen and the proximal end of the bone.
- The site index (SI) of the nutrient foramen.

Data were tabulated using Microsoft Excel. The mean bone lengths and the mean diameter of the NF were compared between left and right limbs for each bone, and the data analyzed using the Kruskal–Wallis test. Statistical analysis was done using Minitab 16.0^®^ statistical software (Minitab Inc., State College, PA). Differences were statistically significant at *p* < 0.05.

## Results

### Direction of the bone and number of the nutrient foramen

**Figure 1.**
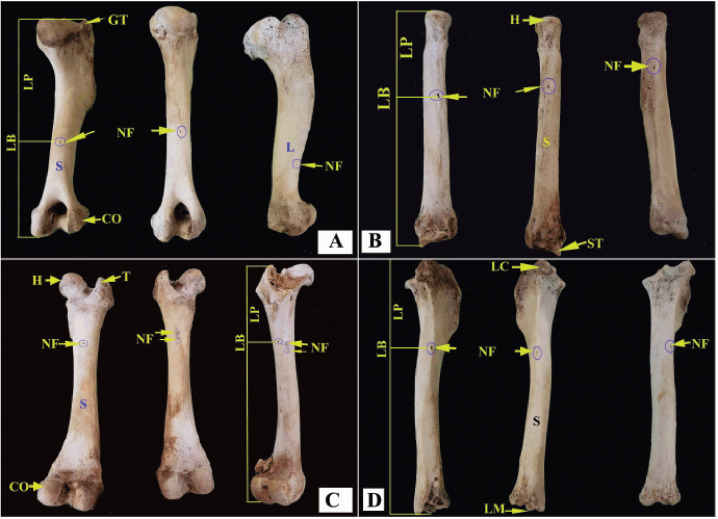
The diaphyseal nutrient foramen in the humerus (A), radius (B), femur (C), and tibia (D) of mixed-breed dogs. CO = Condyle; GT = Greater tubercle; H = Head; L = Lateral surface; LB = Total length of the bone; LC = Lateral condyle; LM = Lateral malleolus; LP = Distance between the NF and the proximal end of the bone; NF = Nutrient foramen; S = Caudal surface; ST = Styloid process.

The nutrient foramen was single in all examined right and left humeri, radii, and tibias. The nutrient foramen was single in all examined right femurs, while it was single in 60%, double in 20%, and triple in 20% of the examined left femurs (Fig. 1 and Table 1)

### Location of the nutrient foramen

The nutrient foramen was localized in the caudal surface in all examined right and left radii, femurs, and tibias. The nutrient foramen was situated in the caudal surface in all examined left humeri, while it was located in the caudal and lateral surfaces of 75% and 25% of the right humeri, respectively (Fig. 1 and Table 1).

### Diameter of the nutrient foramen

The diameter of the nutrient foramen on the humeri ranged from 0.5 to 1.0 mm, with the average diameter being 0.94 (± 0.17) mm. The average diameter of the nutrient foramen on the right humeri was greater than that of the left (1.0 *vs.* 0.88 mm), but this difference was not statistically significant (*p* = 0.39). When evaluating the radius, the diameter of the nutrient foramen ranged from 1 mm to 1.5 mm. The average diameter was 1.1 (± 0.24) mm. Unlike the humerus, the average diameter of the nutrient foramen on the left radius was greater than that of the right (1.25 *vs.* 1.13 mm), and this difference was not statistically significant (*p* = 0.54). Quite interestingly, although the humerus is a larger bone than the radius, the diameter of the nutrient foramen of the humerus was significantly less than that of the radius (0.94 *vs.* 1.1 mm; *p* = 0.04).

Initially, when evaluating the bones of the hindlimbs, we found the diameters of the nutrient foramen of the femur ranged from 0.5 to 2.0 mm, with the average being 1.38 (± 0.56) mm. Although the average diameter of the nutrient foramen on the right femur was greater than that of the left (1.00 *vs.* 0.88 mm), this difference was not statistically significant (*p* = 0.81). The nutrient foramen of the tibias evaluated ranged from 0.5 to 2.5 mm, with the average diameter being 1.00 (± 0.61) mm. Additionally, unlike the femurs assessed, the average diameter of the nutrient foramen of the left tibia was greater than that of the right tibia (1.25 *vs.* 0.75 mm, *p* = 0.34). When comparing the average diameter of a nutrient foramen in the femur versus that of the tibia, the femur had a higher average nutrient foramen diameter (1.25 *vs.* 1.0 mm; *p* = 0.44).

**Table 1.** Number, site, diameter, and penetration direction of the nutrient foramen in the bones of the limbs of mixed breed dogs.

Bone	Direction		Number of NF			Site of NF		Diameter of NF (mm)					Penetration direction of NF	
	Right %	Left%	1%	2%	3%	Caudal surface%	Lateral surface%	0.5%	1%	1.5%	2%	2.5%	Towards joint%	Away from joint%
Humerus	50		100			75	25		100				100	
		50	100			100		25	75				100	
Radius	50		100			100			75	25			75	25
		50	100			100			50	50			100	
Femur	50		100			100		20	40		40		60	40
		50	60	20	20	100		20	40	20	20		80	20
Tibia	50		100			100		50	50				100	
		50	100			100		25	50			25	100	

### Direction of the nutrient foramen

The penetrating direction of the nutrient foramen was towards the elbow joint in all examined right and left humeri, 100% of left radii and 75% of the right radii evaluated. The direction of penetration was away from the elbow joint in 25% of the right radii. The penetration direction of the nutrient foramen was towards the stifle joint in 80% of the left femurs and 60% of the right femurs and all examined right and left tibias, while it was directed away from the stifle joint in 20% and 40% of the left and right femurs, respectively (Tables 1 and 2).

**Table 2.** Bone lengths and the number of NF in the bones of the thoracic limbs of mixed breed dogs.

Classification		Length of bone (cm)	Penetration direction of NF (%)		Diameter of NF (mm)	SI of NF(%)	*p* value
			Towards elbow joint	Away elbow joint			
Humerus	Left (4)	16.93 ± 1.18	100	0	0.88 ± 0.25	64.80	0.04*
	Right (4)	17.55 ± 0.39	100	0	1.00 ± 0.0	51.40	
Radius	Left (4)	18.30 ± 1.50	100	0	1.25 ± 0.29	29.82	0.72
	Right (4)	18.15 ± 1.30	75	25	1.13 ± 0.25	29.47	

* Values are expressed as the mean of a minimum of four bones ± SD.

**Table 3.** Bone lengths and the number of NF in the bones of the hindlimbs of mixed breed dogs.

Classification		Length of bone (cm)	Penetration direction of NF (%)		Diameter of NF (mm)	SI of NF(%)	*p* value
			Towards stifle joint	Away stifle joint			
Femur	Left (5)	19.02 ± 0.61	80	20	1.2 ± 0.57	37.12	0.99
	Right (5)	19.40 ± 0.74	60	40	1.3 ± 0.67	36.86	
Tibia	Left (4)	18.90 ± 1.75	100	0	1.25 ± 0.87	35.16	0.54
	Right (4)	20.03 ± 1.72	100	0	0.75 ± 0.29	33.86	

*Values are expressed as the mean of a minimum of four bones ± SD.

### Bone length and SI

The average length of the right humeri was greater than that of the left humeri (17.55 *vs.* 16.93 cm); however, this difference was not statistically significant (*p* = 0.25). Interestingly, the SI percentage (SI%) for the nutrient foramen on the left humerus was greater than that of the right humerus of the dogs (64.80% *vs.* 51.40%, *p* = 0.04). In contrast to the humerus, the average length of the right radius of the dogs studied was less than that of the left radius (18.15 *vs.* 18.30 cm); however, similar to the humerus, this difference was not statistically significant (*p* = 0.60). The SI percentage (SI%) for the nutrient foramen on the left radius was greater than that of the right radius of the dogs (29.82% *vs.* 29.47%), but unlike as was observed with the humerus, this difference (*p* = 0.72) was found to be not statistically significant (Table 2)

The average length of the right femur was greater than that of the left femur (19.40 *vs.* 19.02 cm); however, this difference was not statistically significant (*p* = 0.40). Interestingly, the SI percentage (SI%) for the nutrient foramen on the left femurs was greater than that of the right femurs (37.12% *vs.* 36.86%; *p* = 0.99). The average length of the right tibia was greater than that of the left tibia of the dogs (20.03 *vs.* 18.90 cm); however, similar to the other bones evaluated in this study, this difference between left and right tibias was not statistically significant (*p* = 0.89). The average SI percentage (SI%) for the nutrient foramen on the left tibia was also greater than that of the right tibia of the dogs (35.16% *vs.* 33.86%), but this difference was found to be not statistically significant (*p* = 0.54) (Table 3).

## Discussion

The nutrient foramen of long bones plays an integral role in bone fracture healing and repair, microvasculature bone surgery, diaphyseal transplantation, and bone grafting [17,29]. Anatomical studies such as this one provide critical information to ensure the nutrient vessels found within this foramen are not compromised during surgical procedures [28]. While the anatomical characterization and localization of NF have been described for humans [7,31-34] and numerous domestic species [4,23,30,35,36], there is a dearth of published information describing this in mixed breed dogs. Despite an extensive literature search, no studies could be found describing the anatomical characterization of the nutrient foramen in mixed breed dogs. 

One nutrient foramen was found in all right and left humeri and the right and left radii of mixed breed dogs. A similar result was observed in the humerus of the dog and Equidae, 98% of large and small ruminants [36], and in 94.7% of the humerus of German Shepherd dogs [4]; however, The German Shepherd dog had two NF in five of the humeri examined [4]. In contrast, it was not found in 1% of the examined humeri in horses, ruminants, pigs, and dogs [36]. In this study, only one nutrient foramen was observed in all the tibias, and right femurs examined, while it was solitary in 60%, double in 20%, and triple in 20% of the left femurs of mixed breed dogs. A similar result was found in the tibias of the German Shepherd dog [30], while it was single in 78.6% and doubled in 21.4% of the femurs of the dog [4], however, the nutrient foramen was solitary in 6.2%, double in 60.8%, triple in 28.9%, and quadruple in 4.4% of the femurs examined in German Shepherd dogs [30]. More than one foramen in the left femurs of the mixed breed dogs could be due to the arterial branches from the proximal, middle, and caudal femoral arteries arterial entering the femur as the primary nutrient artery or as an additional nutrient artery. A similar observation has also been reported in dogs by Cuthbertson *et al.* [37].

The nutrient foramen was located on the caudal surface of all examined humeri of mixed breed dogs; a similar result was reported in dogs [36,38,39], sheep, and pigs [35,40]. However, it should be noted; the nutrient foramen was located either on the caudal surface (73%), cranial surface (14%), or medial surface (13%) of the humeri of German Shepherd dogs [4]. On the other hand, the nutrient foramen was located on the caudal surface in all humeri of pigs, 39% of large ruminants and dogs, and 85% of small ruminants. Comparatively, the nutrient foramen is localized on the medial surface in horses and cats [36]. In this study, the nutrient foramen was located on the caudal surface of all radii examined. A similar observation has been reported in the dog [4,38] and pig [35].

The nutrient foramen was located on the caudal surface of all examined femurs and tibias of mixed breed dogs; a similar result was also reported in other canine studies [10,30,41]. This location is not consistent across species as it was reported to be on the anterior surface of the femur of sheep and pigs [35,40]. This is in contrast to those who reported that although the nutrient foramen was found on the caudal surface of the majority of German shepherd dogs, some also had nutrient foramen on lateral surfaces of their tibias [4].

The range of the diameter value of the nutrient foramen was 0.88–1 mm in the humeri and 1.13–1.25 mm in the radii in mixed breed dogs which are higher than that of German Sphered dog [4], where it was found to be 0.73–0.78 in the humeri and 0.74–0.76 in the radii. Moreover, the diameter of the nutrient foramen of the humeri in mixed breed dogs was 0.5 mm in 25% of the left humeri and 0.1 mm in 100% of the right humerus, and 75% of the left humerus, while it was 1.2 mm in 24%, 0.7 mm in 43%, and 0.55 in 33% of the humerus of the dog; 0.1 in 100% of Carnivora-cats; 0.9 mm in 2%, 0.7 mm in 22%, and 0.55 mm in 76% of small ruminants; 1.2 mm in 83*, and 0.9 mm in 17% of sus; 1.2 mm in 79%, and 0.9 mm in 21% of equidae and 1.2 mm in 76%, and 0.9 mm in 24% of large ruminants [36].

The range of the diameter of the nutrient foramen of the femurs (1.2–1.3 mm) and tibias (0.75–1.25 mm) was higher in mixed breed dogs than that of German Sphered dog [30], where it was found to be 0.45–0.75 in the femurs and 0.78–0.83 mm in the tibias.

The penetration of the nutrient foramen of the humeri of mixed breed dogs was towards the elbow joint. This is because the nutrient artery branched from the collateral radial artery; a similar result was detected in the humeri of German Shepherd dogs [4]. The penetration direction of the nutrient foramen in the radius of mammals has been reported to vary in its direction [30,42]. For example, it is directed towards the elbow joint in the left radii and 75% of the right radii. This direction is due to the nutrient artery for the radius being a branch from the caudal interosseous artery which extends distally in dogs [41], The other 25% of nutrient foramen are directed away from the elbow, and this is potentially due to changes in the direction of the nutrient artery at its origin [17]. The difference in the direction of penetration of the nutrient foramen has also been reported in German Shepherd dogs [4].

The nutrient foramen in the mixed breed dogs used in our study was directed towards the stifle joint in 80% of the left femurs and 60% in the right femurs, while it was directed away from the stifle joint in 40% and 20% of the right and left femurs, respectively. However, the nutrient foramen was directed towards the stifle joint in 49.1% of the right and left femurs, while it was led away from the stifle joint in 50.9% of the right and left femurs in German Shepherd dogs [30]. Moreover, the nutrient foramen was directed towards the stifle joint in all examined tibias, as found in German Shepherd dogs [30]. The direction of the nutrient foramen in most femurs and all of the tibias of mixed breed dogs were directed towards the stifle joint could be due to the medial circumflex femoral and caudal tibial arteries, which are the main arteries of these bones branched above the proximal extremity of the bones and directed distally [23,37].

None of the examined humeri, radii, femurs, and tibias of mixed breed dogs had more than three NF along their diaphysis; a similar result was also reported for German Shepherd dogs [4]. 

The SI percentage (SI%) for the nutrient foramen on the left humeri of mixed breed dogs was greater than that of the right humeri, and this difference was found to be statistically significant (*p* = 0.04); However, these results are in contrast to that of Yılmaz [36] who reported no statistically significant difference in the nutrient foramen of the right and left humerus (*p* > 0.05). Sim and Ahn [4] also reported no difference between the diameter of the nutrient foramen in the right and left humeri and radii of the German Shepherd dog. Similar to our study, the differences in the diameter of the nutrient foramen between left and right humeri were statistically significant (*p* < 0.05) in small ruminants [36]. One limitation of our study is the small number of bones examined. These bones originated from mixed-breed dogs, and there may have anatomical differences in other breeds of dogs. 

## Conclusion

The NF are the major pathways for the entry of the nutrient artery into the diaphysis of long bones in mixed dogs. Knowledge of the amount, location, and direction of the nutrient foramen is essential for evaluating pathological conditions related to these bones and vessel protection during orthopedic procedures. This study showed anatomical differences between breeds of dogs, which must be taken into account when planning and performing orthopedic procedures.

## List of Abbreviations

millimeter, mm; Standard Deviation, SD; Site Index, SI; Trinidad and Tobago, T&T. 

## References

[1] Dai J, Rabie BM (2007). VEGF: an essential mediator of both angiogenesis and endochondral ossification. J Dent Res.

[2] Hankenson KD, Dishowitz M, Gray C, Schenker M (2011). Angiogenesis in bone regeneration. Injury.

[3] Filipowska J, Tomaszewski KA, Niedźwiedzki Ł, Walocha JA, Niedźwiedzki T (2017). The role of vasculature in bone development, regeneration and proper systemic functioning. Angiogenesis.

[4] Sim J, Ahn D (2014). Anatomy of the diaphyseal nutrient foramen in the long bones of the pectoral limb of German Shepherds. Korean J Vet Res.

[5] Uzuner MB, Ocak M, Geneci F, Kocabıyık N, Sargon MF, Al-Shouk A (2018). Quantitative and morphometric evaluation of the foramina nutricia in the long bones of the upper and lower extremities in Anatolian population. Kafkas J Med Sci.

[6] Zahra SU, Kervancioğlu P, Bahşi İ (2018). Morphological and topographical anatomy of nutrient foramen in the lower limb long bones. Eur J Ther.

[7] Kumar K, Mogha IV, Aithal HP, Kinjavdekar P, Amarpal, Singh GR (2007). Occurrence and pattern of long bone fractures in growing dogs with normal and osteopenic bones. J Vet Med A Physiol Pathol Clin Med.

[8] Ljunggren G (1971). Fractures in the dog. A study of breed, sex, and age distribution. Clin Orthop Relat Res.

[9] Singh AP, Mirakhur KK, Nigam JM (1983). A study on the incidence and anatomical locations of fractures in canine, caprine, bovine, equine and camel. Indian J Vet Surg.

[10] Kara ME, Sevil-Kilimci F, Onar V (2011). Foraminal index on the dog femora. Ankara Üniv Vet Fak Derg.

[11] Coolbaugh CC (1952). Effects of reduced blood supply on bone. Am J Physiol.

[12] Laing PG (1956). The arterial supply of the adult humerus. J Bone Joint Surg Am.

[13] Rhinelander FW (1974). Tibial blood supply in relation to fracture healing. Clin Orthop Relat Res.

[14] Welch JA, Boudrieau RJ, DeJardin LM, Spodnick GJ (1997). The intraosseous blood supply of the canine radius: implications for healing of distal fractures in small dogs. Vet Surg.

[15] Daniel A, Read RA, Cake MA (2008). Vascular foramina of the metacarpophalangeal sesamoid bones of Greyhounds and their relationship to sesamoid disease. Am J Vet Res.

[16] Kirschner MH, Menck J, Hennerbichler A, Gaber O, Hofmann GO (1998). Importance of arterial blood supply to the femur and tibia for transplantation of vascularized femoral diaphyses and knee joints. World J Surg.

[17] Kizilkanat E, Boyan N, Ozsahin ET, Soames R, Oguz O (2007). Location, number and clinical significance of nutrient foramina in human long bones. Ann Anat.

[18] Shulman SS (1959). Observations on the nutrient foramina of the human radius and ulna. Anat Rec.

[19] Skawina A, Wyczolkowski M (1987). Nutrient foramen of humerus, radius and ulna in human fetuses. Folia Morphol.

[20] Piermattei DL, Flo GL, Decamp CE (2006). Handbook of small animal orthopedics and fracture repair.

[21] Kara ME, Sevil-kilimci F, Onar V (2011). Foraminal index on the dog femora. Ankara Üniv Vet Fak Derg.

[22] Xue Z, Ding H, Hu C, Xu H, An Z (2016). An anatomical study of the nutrient foramina of the human humeral diaphysis. Med Sci Monit.

[23] Hughes H (1952). The factors determining the direction of the canal for the nutrient artery in the long bones of mammals and birds. Acta Anat (Basel).

[24] Longia GS, Ajmani ML, Saxena SK, Thomas RJ (1980). Study of diaphyseal nutrient foramina in human long bones. Acta Anat (Basel).

[25] Mysorekar VR (1967). Diaphysial nutrient foramina in human long bones. J Anat.

[26] Wade CM (2011). Inbreeding and genetic diversity in dogs: results from DNA analysis. Vet J.

[27] Gümüsburun E, Yücel F, Ozkan Y, Akgün Z (1994). A study of the nutrient foramina of lower limb long bones. Surg Radiol Anat.

[28] Kumar R, Mandloi RS, Singh AK, Kumar D, Mahato P (2013). Analytical and morphometric study of nutrient foramina of femur in Rohilkhand Region. Innovative J Med Health Sci.

[29] Wavreille G, Dos Remedios C, Chantelot C (2006). Anatomic bases of vascularized elbow joint harvesting to achieve vascularised allograft. Surg Radiol Anat.

[30] Ahn D (2013). Anatomical study on the diaphyseal nutrient foramen of the femur and tibia of the German shepherd dog. J Vet Med Sci.

[31] Prashanth KU, Murlimanju BV, Prabhu LV, Kumar CG, Pai MM, Dhananjaya KVN (2011). Morphological and topographical anatomy of nutrient foramina in the lower limb long bones and its clinical importance. Australas Med J.

[32] Ukoha UU, Umeasalugo KE, Nzeako HC, Ezejindu DN, Ejimofor OC, Obazie IF (2013). A study of nutrient foramina in long bones of Nigerians. Natl J Med Res.

[33] Gupta AK, Ambekar MN (2016). Study of nutrient foramina in adult human femur bones. J Nepalgunj Med Coll.

[34] Mishra AK, Jaiswal S, Verma RK, Mishra G, Kumar N (2019). A topographical study of nutrient foramen in dry human long bones of the superior extremity. Era J Med Res.

[35] Payton CG (1934). The position of the nutrient foramen and direction of the nutrient canal in the long bones of the Madder-fed pig. J Anat.

[36] Yılmaz O (2020). Anatomic characteristics and locations of nutrient foramen in humerus of domestic animals. Turk J Vet Res.

[37] Cuthbertson EM, Gilfillan RS (1964). Variations in the anatomic origin of the nutrient artery of the canine femur. Anat Rec.

[38] Jojic D, Blagojevic Z (1983). Nutrient foramina on the bones of the front extremities in dogs. AGRIS.

[39] Miller ME, Christensen GC, Evans HE (1964). Anatomy of the dog.

[40] Johnson V, Beckett S, Marquez-Grant N (2017). Differentiating human versus non-human bone by exploring the nutrient foramen: implications for forensic anthropology. Int J Legal Med.

[41] Evans HE, de Lahunta A (2013). Miller’s anatomy of the gog.

[42] Humphry GM (1861). Observations on the growth of the long bones and of stumps. Med Chir Trans.

